# Validation of the Role of Thrombin Generation Potential by a Fully Automated System in the Identification of Breast Cancer Patients at High Risk of Disease Recurrence

**DOI:** 10.1055/s-0040-1722609

**Published:** 2021-02-10

**Authors:** Patricia Gomez-Rosas, Marina Pesenti, Cristina Verzeroli, Cinzia Giaccherini, Laura Russo, Roberta Sarmiento, Giovanna Masci, Luigi Celio, Mauro Minelli, Sara Gamba, Carmen Julia Tartari, Carlo Tondini, Francesco Giuliani, Fausto Petrelli, Andrea D'Alessio, Giampietro Gasparini, Roberto Labianca, Armando Santoro, Filippo De Braud, Marina Marchetti, Anna Falanga, on behalf of the HYPERCAN Investigators

**Affiliations:** 1 Immunohematology and Transfusion Medicine, Hospital Papa Giovanni XXIII, Bergamo, Italy; 2 Hematology Service, Hospital General Regional Tecamac, IMSS, Estado de Mexico, Mexico; 3 Oncology Unit, Hospitals San Filippo Neri and San Giovanni Addolorata, Rome, Italy; 4 Medical Oncology and Hematology, IRCCS Humanitas Institute, Rozzano, Italy; 5 Medical Oncology and Hematology, IRCCS National Cancer Institute, Milan, Italy; 6 Oncology Unit, Hospital Papa Giovanni XXIII, Bergamo, Italy; 7 Medical Oncology Unit, IRCCS Istituto Tumori Giovanni Paolo II, Bari, Italy; 8 Oncology Unit, Hospital Treviglio-Caravaggio, Treviglio, Italy; 9 Department of Medicine, Gruppo San Donato, Policlinico San Marco, Bergamo, Italy; 10 Department of Oncology Bergamo Province, Hospital Papa Giovanni XXIII, Bergamo, Italy; 11 School of Medicine, University of Milan Bicocca, Italy

**Keywords:** breast cancer, disease recurrence, hypercoagulability, risk model, thrombin generation

## Abstract

**Background** The measurement of thrombin generation (TG) potential by the calibrated automated thrombogram (CAT) assay provides a strong contribution in identifying patients at high risk of early disease recurrence (E-DR). However, CAT assay still needs standardization and clinical validation.

**Objective** In this study, we aimed to validate the role of TG for E-DR prediction by means of the fully automated ST Genesia system.

**Methods** A prospective cohort of 522 patients from the HYPERCAN study with newly diagnosed resected high-risk breast cancer was included. Fifty-two healthy women acted as controls. Plasma samples were tested for protein C, free-protein S, and TG by ST Genesia by using the STG-ThromboScreen reagent with and without thrombomodulin (TM).

**Results** In the absence of TM, patients showed significantly higher peak and ETP compared with controls. In the presence of TM, significantly lower inhibition of ETP and Peak were observed in patients compared with controls. E-DR occurred in 28 patients; these patients had significantly higher peak and endogenous thrombin potential (ETP) in the absence of TM compared with disease-free patients. Multivariable analysis identified mastectomy, luminal B HER2-neg, triple negative subtypes, and ETP as independent risk factors for E-DR. These variables were combined to generate a risk assessment score, able to stratify patients in three-risk categories. The E-DR rates were 0, 4.7, and 13.5% in the low-, intermediate-, and high-risk categories (hazard ratio = 8.7; *p* < 0.05, low vs. high risk).

**Conclusion** Our data validate the ETP parameter with a fully automated standardized system and confirm its significant contribution in identifying high-risk early breast cancer at risk for E-DR during chemotherapy.

## Introduction

Thromboembolic disease is one of the most common comorbidities associated with cancer, representing the second leading cause of mortality after malignant disease itself. 1 2 In cancer patients, thrombosis can be considered the clinical manifestation of a deeper and systemic activation of the hemostatic system, occurring in almost all types of cancer. 3 This coagulopathy recognizes multiple pathogenetic mechanisms, including the prothrombotic properties expressed by tumor tissues and anticancer therapies, altogether able to activate different hemostatic compartments of the host. 3 4 5

Interestingly, cancer-associated hypercoagulability not only promotes thromboembolism but also drives proliferation and dissemination of the malignant disease. 6 7 In addition, this cancer-associated coagulopathy worsens with the progression of the malignant disease, sustaining a tight connection between tumor burden and blood clotting deregulation. 8

Based on this relationship, in the attempt to identify more accurate prognostic cancer biomarkers, in the last decades several studies analyzed different hemostatic biomarkers in relation to specific cancer outcomes, including disease progression, treatment response, and mortality. 9 10 11 12 13 14 Along with this line of research, in 2012 our group launched the HYPERCAN (HYPERcoagulation in CANcer) study, an ongoing prospective observational Italian multicenter study, specifically designed to assess whether the occurrence of a hypercoagulable state may be predictive for cancer disease outcome and thrombosis in patients with different types of cancer. 15

Recently, in the frame of the HYPERCAN study, we could demonstrate in a cohort of high-risk breast cancer patients with surgically removed tumor and candidate to receive adjuvant chemotherapy, a crucial role of thrombin generation (TG) performed by the calibrated automated thrombography (CAT) in identifying subjects at high risk of disease relapse. 16 The CAT is a global hemostatic assay that represents an in vitro test that evaluates the capacity of an individual to generate thrombin resulted from the relative strength of the pro- and anticoagulant drivers. TG can detect coagulation factor deficiencies; it is sensitive to anticoagulant drugs and to prothrombotic states such as deficiency of antithrombin, protein C (PC), or protein S as well as to hyperprothrombinemia and resistance to activated PC. 17 Particularly, in our previous study, among the different TG parameters, the endogenous thrombin potential (ETP), defined as the net amount of thrombin that plasma can generate, was successfully integrated in a prediction risk assessment model, ameliorating the identification of subjects at higher risk of early relapse (i.e., within 2 years). 16

TG by the CAT assay is well reproducible within a single laboratory when standardized preanalytical and analytical conditions for measurement are applied. 18 19 20 However, partial automation, lack of reagent standardization, and clinical validation limit the use of CAT assay in the clinical practice. 21 22 23 24 Recently, a new TG analyzer (ST Genesia, Stago) has been released as the first fully automated TG system for clinical routine laboratories together with a set of reagents balanced for sensitivity to procoagulant and anticoagulant protein deficiencies. 25 For example, by performing the TG assay in the presence of thrombomodulin (TM) with the STG-ThromboScreen assay, it is possible to detect impairments in the natural anticoagulant PC system that prone to a hypercoagulable state. The clinical application of ST Genesia has been evaluated until now in the setting of anticoagulation, 26 27 28 liver diseases, 29 30 31 32 and diabetes. 33 In the present study, for the first time, the ST Genesia was tested in a prospective cohort of breast cancer patients with the aim to validate the role of TG in identifying patients at high risk of early cancer relapse and detect underlying mechanisms of cancer-associated hypercoagulability.

## Methods

### Study Cohort and Follow-Up

The study included female patients enrolled in the ongoing, multicenter, and prospective HYPERCAN study. 15 The inclusion criteria were having surgically resected breast cancer with a stage of T2-T4 N0 M0 or TX N+ M0 (TNM classification) and being candidate to adjuvant systemic chemotherapy for the high-risk profile of the tumor. Patients with acute medical illness, terminal conditions or hospitalized, under therapeutic anticoagulant therapy, with recent thrombosis or life expectancy less than 3 months were not eligible. Data on the medical history and tumor characteristics were collected, and all participants gave their written informed consent. The local ethics committee approved the study protocol, according to the last revision of the Helsinki Declaration. Patients were categorized in subtypes according to tumor expression of estrogen receptor (ER), progesterone receptor (PR), Ki67, and HER2, as per the ASCO-CAP guidelines. 34 The following molecular subgroups were identified: luminal A (ER/PR positivity, HER2 negativity, low Ki67), luminal B HER2-neg (ER/PR positivity, HER2 negativity, high Ki67), luminal B HER2-pos (ER/PR positivity, HER2 positivity, any ki67), HER2-positive (ER/PR negativity, HER2 positivity), and triple negative (ER/PR and HER2 negativity). After enrollment, patients were followed-up annually for a maximum of 5 years. At each follow-up visit, clinical information on any antitumor treatment, and disease recurrence (DR) were recorded.

### Blood Sampling and Plasma Preparation

For coagulation assays, fasting peripheral venous blood samples were collected with a 21-gauge needle, after discarding the first 2 to 3 mL of blood, into vacutainer tubes containing 0.109 M sodium citrate (9:1 vol/vol; Becton Dickinson, Vacutainer, Plymouth, United Kingdom). The preanalytical phase consisted in two sequential centrifugations at 2,600 rpm for 15 minutes at 25°C, and isolated plasma was aliquoted and stored at −80°C. Plasma samples were thawed 5 minutes in a 37°C water bath, homogenized, and stored at room temperature for a maximum of 20 minutes before starting the tests. All samples were tested at the Laboratory of Hemostasis and Thrombosis Center (Hospital Papa Giovanni XXIII, Bergamo, Italy). Blood sampling, plasma preparation, and storage have been conducted according to standardized procedure and international recommendations. 22 35

### Thrombin Generation Assays

TG potential was investigated in plasma samples by the ST Genesia Thrombin Generation System (Stago, France), according to the manufacturer's recommendations. The ST Genesia system is a dedicated platform to enable fully automated quantitative and standardized evaluation of TG by using dedicated reagents, calibrator, quality controls, and reference plasmas. Specifically, for the present study, TG was performed in the absence (–TM) or presence of TM (+TM) by using the STG-ThromboScreen reagent. According to the information released by the manufacturer, this reagent contains a mixture of phospholipids, recombinant human TF at an intermediate picomolar concentration, and TM at a concentration able to inhibit 50% of the ETP obtained in the absence of TM on normal plasma. The specific concentrations of TF, phospholipids, and TM are not disclosed by the manufacturer. On each day of testing, a new calibration test with three levels of quality control (low, normal, and high), and a reference plasma to normalize parameters of TG performed in the absence of TM. The normalization aims to reduce the interlaboratory variability as well as the variability between different measurement runs. TG started by dispensing a mixture of calcium chloride and a Z-Gly-Gly-Arg-AMC fluorogenic substrate (STG-FluoStart), and calibration was compensated for each sample by using a known amount of AMC fluorogenic marker (STG-FluoSet). The following parameters of TG curve were considered: lag time (in minutes: time from test triggering to signal detection), time to peak (ttP; in minutes: time necessary for thrombin concentration to reach its maximal value), peak height (in nM: maximal thrombin concentration), and ETP (endogenous thrombin potential, in nM.min: area under the thrombin time concentration curve). Normalized parameters of TG − TM are expressed as “ratio” for lag time and ttp, and as “percentage” for TG peak and ETP. In addition, the ETP inhibition (ETP − Inh) = ([ETP − TM] – [ETP + TM]/ETP − TM) and peak inhibition (peak − Inh) = ([Peak − TM] – [Peak + TM]/Peak − TM) were calculated and used to estimate the degree of function of PC anticoagulant pathway.

Tests for inter- and intra-assay coefficients of variation (CV) of TG parameters were performed by two different operators by using a normal pooled plasma (NPP); for the interassay variability, 24 independent assays (each with a different calibration curve and different controls) were performed while, for the intra-assay variability, NPP was measured in a single run 17 times. The inter- and intra-assay CVs for absolute parameters were 3.65 and 2.64% for lag time, 3.39 and 2.23% for ttP, 6.47 and 3.36% for ETP, and 5.62 and 2.01% for peak. For normalized parameters, the inter- and intra-assay CVs were 4.63 and 2.61% for lag time, 4.32 and 2.27% for ttP, 4.06 and 3.37% for ETP, and 6.04 and 2.74% for peak. Control samples were obtained from 52 female hospital employees and served for the laboratory tests. Subjects were free of cardiovascular disease, thrombotic or bleeding disorders, diabetes, cancer, or infectious diseases, and were not taking antiplatelet or anti-inflammatory drugs in the last 10 days before blood sampling. Subjects on anticoagulants were not included. ETP reference intervals were defined as the 5th and 95th percentiles of the ETP values obtained in healthy controls' plasma samples. Hypercoagulable laboratory phenotype was defined by an ETP value above the upper limit of the reference intervals.

### Anticoagulant Proteins

Plasma levels of PC (HemosIL Protein C, Werfen Group) and Free-protein S (HemosIL Free Protein S, Werfen Group) were measured on an automated coagulometer analyzer (ACL TOP500, Werfen Group).

### Study Outcome

In the present study, the primary outcome is the occurrence of early disease recurrence (E-DR), defined as either loco-regional (limited to the ipsilateral breast or chest wall and/or axillary, infraclavicular, or supraclavicular lymph nodes) or distant metastasis within 2 years from starting chemotherapy. For descriptive analysis, patients were divided according to the primary clinical outcome: subjects with early relapse (E-DR) and subjects who remained 2-year disease-free (DF).

### Statistical Analysis

In the descriptive statistics, categorical data were summarized as frequencies and proportions. Continuous variables were summarized as median and 5th to 95th percentile range (95% confidence interval [CI]). Differences between groups were tested by Pearson's Chi-square or Mann–Whitney *U* -test. Association between variables was estimated by the Spearman correlation coefficient (ρ). Survival analysis was done by the Kaplan–Meier and Cox proportional hazard models. The multivariable Cox regression analysis was performed by a backward variable selection including clinical (body mass index [BMI], smoke, infection, relevant comorbidities, concomitant use of anticoagulant/antiplatelet agents, presence of central venous catheter (CVC), tumor molecular subtype, tumor size, lymph node status, tumor grading, Eastern Cooperative Oncology Group Performance Status [ECOG-PS], antitumor therapy, and type of surgery), and laboratory covariates (ETP and peak of TG in the presence and absence of TM, ETP and peak inhibition, PC and free-PS). Statistical analysis has been performed by using SPSS v26.0 (IBM Corp) and the Prism software version 8 (GraphPad software, Inc).

## Results

### Characteristics of the Study Population

Characteristics of the study population are depicted in Table 1 . A total of 522 female patients with a median age of 50 years (95% CI: 31–71 years) were analyzed. Breast-conserving resection was performed in 59%, and mastectomy in 41% of them. HER-2 expression was positive in 149 and negative in 373 patients' specimens. The study population was classified in five different groups according to the molecular subtypes: luminal B HER2-neg was the most frequent (33%) followed by luminal A (22.6%), luminal B HER2-pos (19.3%), TN (15.9%), and HER2-pos (9.2%).

**Table 1:** Baseline characteristics of the overall cohort of patients and according to disease recurrence

	All patients ( *n* = 522)	E-DR ( *n* = 28)	DF ( *n* = 494)	*p* -Value
**Age (y [median/5–95th])**	50 (31–71)	49 (37–77)	52 (36–72)	0.788
**Molecular subtype (%)**				
Luminal A	118 (22.6)	4 (14.3)	114 (23.1)	0.014
Luminal B HER2-neg	172 (33)	11 (39.3)	10 (35.7)	
Luminal B HER2-pos	101 (19.3)	1 (3.6)	100 (20.2)	
HER2-pos	48 (9.2)	2 (7.1)	46 (9.3)	
Triple negative	83 (15.9)	10 (35.7)	73 (14.8)	
**Surgery (%)**				
Breast conserving	308 (59)	14 (50)	294 (60)	0.242
Mastectomy	214 (41)	14 (50)	200 (40)	

Abbreviations: DF, disease free; E-DR, early disease recurrence; ETP, endogenous thrombin potential; HR, hazard ratio.

Note: Data are presented as number (percentage). *P* is statistical significance by Pearson's Chi-square test (or by Mann–Whitney test for age) for comparison between patients with early disease recurrence and 2-year disease free.

### Thrombin Generation Potential by ST Genesia

Results of TG potential performed in the absence and in the presence of TM in the overall cohort of patients and controls are described in Table 2 . Data for each TG parameter are reported both in absolute and normalized values. In the absence of TM (-TM), ttP was significantly ( *p* < 0.001) shorter in patients compared with controls, with no differences in lag time. Additionally, patients exhibited significantly ( *p* < 0.001) higher values of ETP, and thrombin peak compared with controls, both as absolute and normalized values. According to 95th percentiles of the ETP distribution in the control group of heathy subjects, 14% of the patients presented a hypercoagulable laboratory phenotype (i.e., ETP > 1,591 nM.min).

**Table 2:** Thrombin generation parameters obtained in the absence and presence of thrombomodulin in patients compared with controls

	TG − TM		*p-* Value	TG + TM		*p-* Value
	Controls *n* = 52	Patients *n* = 522		Controls *n* = 52	Patients *n* = 522	
Lag time				Lag time		
Min Ratio	1.97 (1.59–3.04) 1.17 (0.92–1.81)	1.97 (1.53–2.81) 1.17 (0.91–1.67)	0.473 0.481	2.29 (1.82–3.56) ^a^	2.23 (1.71–3.20) ^a^	0.064
Time to peak				Time to peak		
Min Ratio	4.07 (3.44–5.83) 1.19 (0.97–1.71)	3.85 (3.02–5.20) 1.11 (0.87–1.51)	0.001 0.001	4.01 (3.40–5.57) –	3.88 (3.21–5.14) –	<0.001
Peak				Peak		
nM (%)	260 (160–337) 80.0 (47.8–105)	304 (202–404) 97.6 (61.6–128)	<0.001 <0.001	146 (53.8–241) ^b^ –	205 (91.9–339) ^b^ –	0.039
ETP				ETP		
nM.min (%)	1273 (995–1591) 81.6 (62.4–103)	1354 (977–1824) 91.1 (67.5–122)	0.010 <0.001	581 (219–910) ^b^ –	817 (393–1345) ^b^ –	<0.001

Abbreviations: TG, thrombin generation; TM, thrombomodulin.

Note: Data are reported as median and range (5th–95th percentiles). In the absence of thrombomodulin (−TM), non-normalized and normalized values are provided for each TG parameter. In presence of thrombomodulin (+TM) TG parameters are provided only as absolute values.

a *p* < 0.05.

b *p* < 0.001 versus respective TM values.

**Fig. 1:**
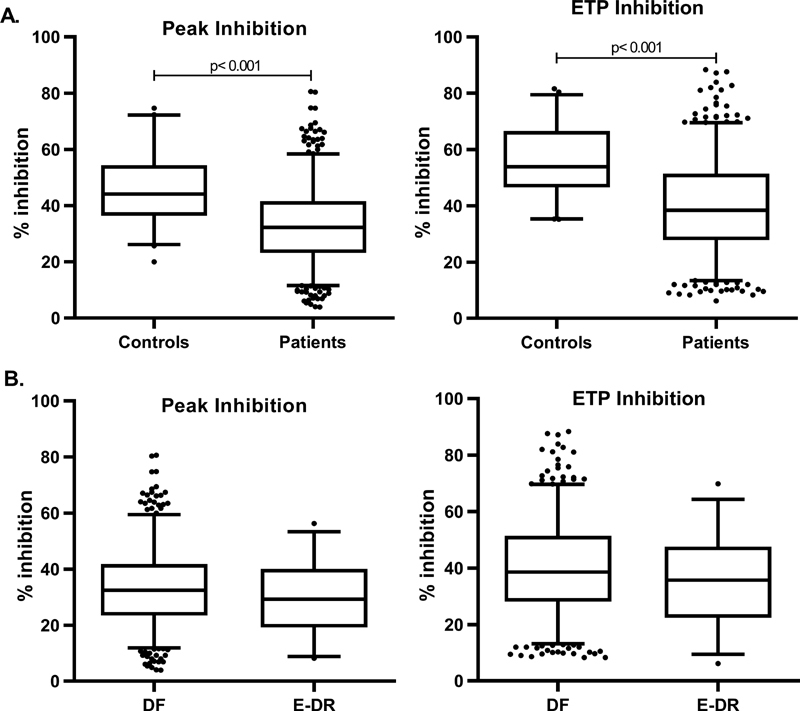
Endogenous thrombin generation potential inhibition and peak inhibition ( **A** ) in patients compared with controls and ( **B** ) in patients with E-DR compared with DF. E-DR, early disease recurrence; DF, disease free.

In the presence of TM, a significant reduction of ETP and peak height was observed compared with the respective values obtained in the absence of TM in both controls and patients. Specifically, the median ETP levels decreased from 1,273 to 581 nM.min in controls ( *p* < 0.01), and from 1,354 to 817 nM.min ( *p* < 0.01) in the cohort of patients. Similar inhibition profiles were obtained with peak height values. The calculation of TG inhibition by TM, showed that the percentage of ETP (38 vs. 50%; *p* < 0.001) and peak inhibition (32 vs. 40%; *p* < 0.001) was significantly lower in the group of patients compared with controls ETP ( Fig. 1A ).

In the overall group of patients, plasma levels of anticoagulant PC and free-PS activity were 118% (95% CI: 90–158) and 84.5% (95% CI: 60.3–118), respectively. No significant differences were observed in PC and PS concentrations between E-DR and DF patients. Slightly but significant associations were observed between ETP inhibition and peak inhibition with protein C (ρ = 0.297; *p* < 0.001 and ρ = 0.335; *p* < 0.001, respectively), and with protein S (ρ = 0.148; *p* = 0.001 and ρ = 0.180; *p* < 0.001, respectively).

### Thrombin Generation Parameters According to Early Disease Recurrence

After surgery, all patients initiated antitumor therapy, which consisted in anthracycline-based regimen in 32.5%; anthracycline plus taxanes to in 55.2%, taxanes-based regimen without anthracyclines in 7.7%; and CMF (cyclophosphamide, methotrexate, 5-fluorouracil) in the remaining 4.6%. All of 149 HER-2 positive patients received (in addition to chemotherapy) trastuzumab every 21 days for 1 year.

After a median follow-up of 3.4 years (95% CI: 1.1–5.3 years), 28 patients experienced a radiographically documented E-DR ( Table 1 ), representing the 5.36% of the study population. Patients with E-DR showed a significant different distribution of molecular subtypes ( *p* = 0.014) compare with patients who remained (DF), with a higher frequency of luminal B HER2-neg and TN subtypes in E-DR compare with DF patients. No difference was found in type of surgery according to DR.

**Fig. 2:**
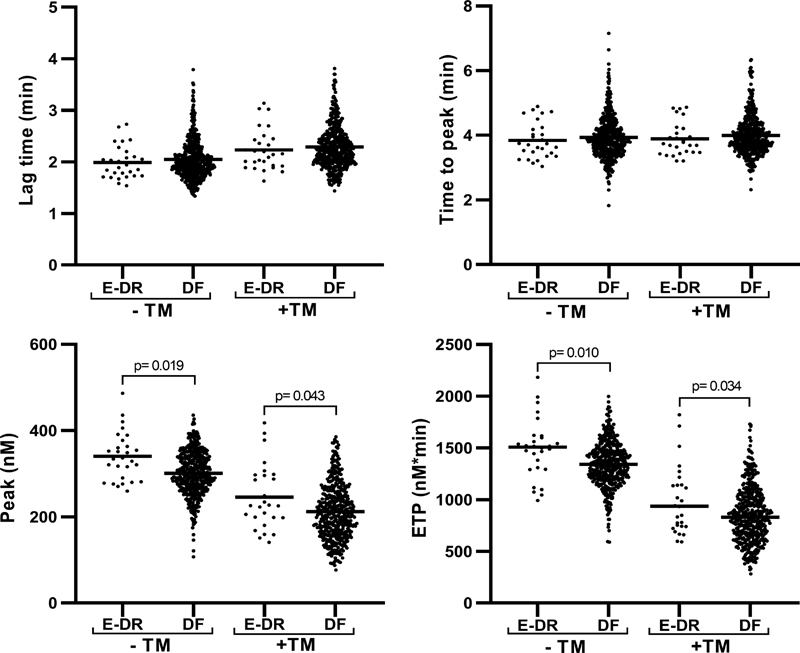
Thrombin generation parameters in the absence and presence of thrombomodulin according to early disease recurrence. ETP, endogenous thrombin potential; TM, thrombomodulina; E-DR, early disease recurrence; DF, disease free.

Fig. 2 shows TG parameters both in the absence and presence of TM according to E-DR. Results showed that lag time and ttP were not statistically different between patients who experienced an E-DR compared with DF patients, while ETP and peak were significantly ( *p* < 0.05) higher in the group of E-DR patients with both −TM and +TM measurements. Differently, no differences were found in the TG parameters ETP − Inh and peak − Inh values according to E-DR ( Fig. 1B ). The univariate Cox regression analysis showed that an increase of ETP value was associated with a higher E-DR risk.

### Endogenous Thrombin Potential-Based Assessment Score for the Prediction of Early Disease Recurrence

**Fig. 3:**
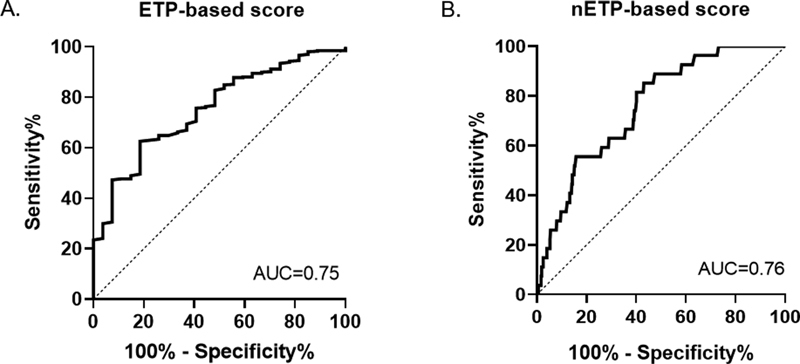
Receiver operating characteristic curve analysis for ETP-based score ( **A** ) and nETP-based score ( **B** ). ETP, endogenous thrombin potential.

**Fig. 4:**
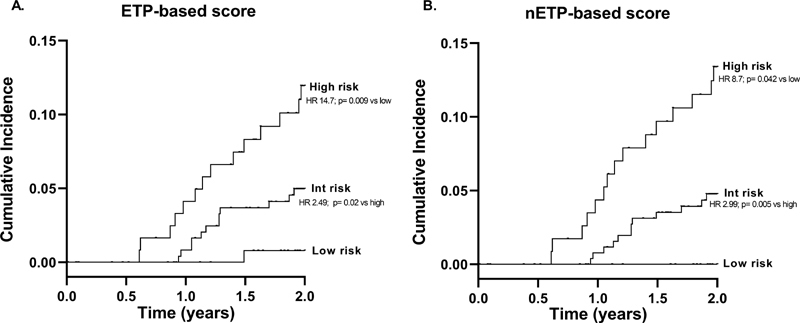
Cumulative incidence of E-DR after curative surgery for breast cancer stratified by ETP-based score ( **A** ) and nETP-based score ( **B** ). (A) The E-DR rates by the ETP-based score were 0.8, 4.9, and 11.8% in the low-, intermediate-, and high-risk categories. Low versus high risk: HR = 14.7 (95% CI: 1.94–112; *p* = 0.009); Intermediate versus high risk: HR = 2.49 (95% CI: 1.15–5.39; *p* = 0.020. (B) The E-DR rates by the nETP-based score were 0, 4.7, and 13.5% in the low-, intermediate-, and high-risk categories. Low versus high risk: HR = 8.70 (95% CI: 1.08–70; *p* = 0.042); intermediate versus high risk: HR = 2.99 (95% CI: 1.40–6.41; *p* = 0.005). CI, confidence interval; E-DR, early disease recurrence; ETP, endogenous thrombin potential; HR, hazard ratio.

A multivariable Cox regression analysis was performed in the cohort of patients starting from a full model including laboratory (i.e., TG ± TM parameters, ETP and peak inhibition, PC, free- PS) and clinical-pathological covariates (i.e., BMI, smoke, infection, relevant comorbidities, concomitant use of anticoagulant/antiplatelet agents, presence of central venous catheter (CVC), tumor molecular subtype, tumor size, lymph node status, tumor grading, Eastern Cooperative Oncology Group Performance Status [ECOG-PS], antitumor therapy, and type of surgery). After backward selection, the multivariable model identified ETP value (both absolute and normalized), mastectomy, luminal B HER2-neg, and TN subtypes as independent risk factors for E-DR. The coefficients of the four variables provided by this multivariable analysis were combined in a formula for the generation of a linear risk score. Specifically, two scores were generated: one including ETP absolute value (ETP-based score) and the other including the corresponding normalized ETP value (nETP-based score). As shown in Table 3 , the ETP score was based on the following formula: (ETP × 0.002) + 0.998 (if luminal B HER2-neg) + 1.597 (if TN) + 0.857 (if mastectomy) ( Table 3 ), while nETP-based score was as follows: (nETP × 0.035) + 1.004 (if luminal B HER2-neg) + 1.627 (if Triple negative) + 0.844 (if mastectomy) ( Table 3 ). The ROC curve analysis was performed to evaluate the accuracy of each score as a continuous variable to predict E-DR. The area under the curve (AUC) was 0.75 for ETP-based score ( Fig. 3A ) and 0.76 for nETP-based score ( Fig. 3B ). The 25th and 75th percentiles of the two prognostic indices were then utilized as cut-off values for the creation of three risk groups categories. In the ETP-based score, low-risk patients had a value <3.1 points, intermediate 3.1 to 4.2 points, and high risk had a score >4.2 points. For nETP-based score, low-risk patients had a value <3.5 points while high-risk group had a score >4.8 points. The Kaplan–Meier curves for both scores according to risk groups are shown in Fig. 4 . ETP-based score provided a cumulative incidence of E-DR equal to 0.8% (95% CI: 0.7–2.3%), 4.9% (95% CI: 2.2–7.7), and 11.8% (95% CI: 6.1–17.9%) in the low-, intermediate- and high-risk groups, respectively (HR: 14.7 [95% CI: 1.94–112]; *p* = 0.009 high vs. low risk; HR: 2.49 [95% CI: 1.15–5.39]; *p* = 0.02 high vs. intermediate risk). The cumulative incidence of E-DR by nETP-based score was 0% in the low risk, 4.7% (95% CI: 2.1–7.2%) in the intermediate risk and 13.5% (95% CI: 7.2–19.8%) in the high-risk category (HR: 8.70 [95% CI: 1.08–70]; *p* = 0.042 high vs. low risk; HR: 2.99 [95% CI: 1.40–6.41]; *p* = 0.005 high vs. intermediate risk). Multivariable cox regression analysis without ETP identified the molecular subtype triple negative (TN) as the only predictive variable statistically associated with E-DR, with a cumulative incidence of E-DR in the TN group of 12.5% (95% CI: 5.3–19.7%) versus 4.3% (95% CI: 2.3–6.3) in the remaining patients.

**Table 3:** Cox regression multivariable analysis for early disease recurrence including absolute and normalized endogenous thrombin potential values

	HR	95% CI	Coefficient	95% CI	*p* -Value
ETP (nM ^∗^ min)	1.002	(1.001–1.004)	0.002	(0.001–0.004)	0.001
Luminal B HER2-neg	2.713	(1.045–7.046)	0.998	(0.044–1.952)	0.040
Triple negative	4.941	(1.797–13.58)	1.597	(0.586–2.608)	0.002
Mastectomy	2.355	(1.062–5.223)	0.857	(0.060–1.653)	0.035
	**HR**	**95% CI**	**Coefficient**	**95% CI**	***p*** - **Value**
nETP (%)	1.035	(1.014–1.058)	0.035	(0.014–0.056)	0.001
Luminal B HER2-neg	2.730	(1.050–7.096)	1.004	(0.049–1.959)	0.039
Triple negative	5.087	(1.848–14.01)	1.627	(0.614–2.639)	0.002
Mastectomy	2.326	(1.052–5.141)	0.844	(0.051–1.637)	0.036

Abbreviations: CI, confidence interval; ETP, endogenous thrombin potential; HER2, human epidermal growth factor receptor 2; nETP, normalized endogenous thrombin potential.

## Discussion

Despite the improvement in the diagnostic and therapeutic armamentarium, breast cancer patients still remain at significant risk of DR in the follow-up after surgery and adjuvant chemotherapy. 36 Predicting the pattern of recurrence is therefore an important issue in this setting, as it can aid in the development of targeted surveillance and treatment strategies.

In our prospective cohort of high-risk surgically resected breast cancer patients, we found a cumulative incidence of DR of 9.8% after a median follow-up of 3.4 years. 12 At 2 years of follow-up, the cumulative incidence was 5.6%, providing the rationale for ameliorate the identification of subjects with worse prognosis, among these high-risk patients.

According to the HYPERCAN main study proposal, we performed a series of prechemotherapy evaluation of different hemostatic biomarkers that allowed us to identify and internally validate a risk assessment model of E-DR based on ETP obtained with the CT assay. 16 However, partial automation, lack of reagent standardization, and clinical validation limit the use of CT assay in the clinical practice, 20 and this prompted us to reevaluate in this cohort patients, the role of ETP by performing the TG analysis by the fully automatized ST Genesia system. In this re-evaluation, we also included the study of the anticoagulant C pathway by the same system with dedicated reagents to further explore the hypercoagulable profile of these patients. Due to the complexity encompassed by cancer-associated coagulopathy, the evaluation of both pro- and anticoagulant forces is of great interest.

In the present study, when compared with controls, high-risk breast cancer patients showed a shift toward a procoagulant direction, as demonstrated by elevated ETP and peak values. Similar results were obtained in our previous study, in which TG measurement was performed by CT assay where patients displayed higher ETP and peak values compared with healthy controls. 16 The differences in ETP and peak values between patients and controls were 6.4 and 17%, respectively, in the absence of TM; the presence of TM magnified these differences that became equal to 40% for both ETP and peak values. The calculation of ETP and peak inhibition by TM, based on values obtained in the presence and absence of TM, provides an estimate of PC anticoagulant pathway function. In this setting, in the healthy control group, we observed a 50% inhibition of ETP by TM, in agreement with manufacturer's information, and published data. 27 Otherwise, significantly lower ETP and peak inhibition were observed in the group of patients compared with controls. The reduced ability of TM to inhibit the TG potential (TM resistance) might underline a possible dysfunction of the PC-anticoagulant pathway in the group of patients. PC is a natural coagulation inhibitor which is activated in vivo by thrombin bound to TM, its endothelial receptor. 37 Once activated, PC degrades FVa and FVIIIa, thus inhibiting further generation of thrombin. In the TG assay performed with exogenous TM, PC is activated as soon as thrombin-TM complex is formed, diminishing TG as shown by the reduction in ETP and peak values. Moreover, to understand whether the dysfunction of the PC-anticoagulant pathway was associated to plasma PC and free-PS concentrations, the levels both anticoagulant proteins were measured in the cohort of patients and analyzed in association with TG parameters. Results showed that anticoagulant proteins levels were significantly correlated with ETP and peak inhibition. However, the low values of correlation coefficients did not allow us to establish a preeminent contribution of protein C and protein S in TM resistance by the TG Genesia assay. Interestingly, dysfunction of the PC pathway in breast cancer patients have been reported by several studies, mainly based on the CT assay performed in the presence of activated PC (APC), and defined as “APC resistance” 38 39 40 . Specifically, in the study by Tinholt et al, 41 in 358 breast cancer patients, individual coagulation factors/inhibitors influencing the acquired resistance to aPC was investigated. The data showed that the main determinants of APC resistance in these patients were fibrinogen (β = −0.35, *p* < 0.001), protein S (β = −0.33, *p* < 0.001), and protein C (β = 0.28, *p* < 0.001) followed by FIX (β = 0.22, *p* = 0.026), and TFPI (β = −0.19, *p* = 0.021). However, the regression models showed that the phenotypic plasma determinants explained modestly 25 and 31% of the variability in APC resistance, suggesting, as in our study, that there are still unidentified causes to be uncovered.

The occurrence of a hypercoagulable state in resected breast cancer patients was demonstrated in our previous study by elevation of some coagulation biomarkers as fibrinogen, F1 + 2, and D-dimer. 12 In the present study, an additional contribution of PC anticoagulant pathway dysfunction to the hypercoagulable phenotype was also demonstrated. 42

The role of TG parameters as prognostic biomarkers in breast cancer has been previously demonstrated in a case–control study where a decrease of lag time and ttP and an increase of peak value predicted the presence of breast cancer. 39 Recently, our group identified and internally validate the role of ETP when included in a risk assessment model for DR. 16 To confirm the relevance of our previous observation, prechemotherapy parameters of TG by ST Genesia were tested in relation to E-DR. Despite the low absolute differences and the large confidence intervals, the comparison of TG parameters between E-DR and DF patients showed significantly elevated ETP and peak values compared with DF subjects, with no differences in ETP and peak inhibition by TM. By the multivariable analysis, ETP values (absolute and normalized values), TN and luminal B HER2-neg molecular subtypes, and mastectomy were identified as independent risk factors for E-DR. The coefficient of these parameters, then provided by the four variables in the multivariable analysis, were utilized to generate two risk assessment scores: one based on ETP absolute values and the second on nETP values. Both scores showed a similar and good precision, providing both an AUC of approximately 0.76. According to selected cut-off values, both scores well stratified the patients in three risks categories, with better discrimination between low- and high-risk categories reached by the nETP-based model (0 vs. 13.5%, HR: 8.7). Without the inclusion of ETP, the only clinical-pathological variable associated to E-DR was the TN molecular subtype, that, as single variable did not allow for a stratification of the risk comparable to that obtained with our ETP-based model. Indeed, compared with the stratification of patients according to being or not TN, with the ETP-based score we improved the discrimination as we obtained a low-risk group with a cumulative incidence of 0.8% for absolute ETP and 0% for nETP. Furthermore, the number of subjects in the high-risk category increased.

A possible study limitation may consist in the global evaluation of TG that does not allow distinguishing the contribution of any single factor in determine clotting system imbalance. Nevertheless, the overall representation of procoagulant and anticoagulant forces provided by TG assay could be regard as an advantage as it constantly represents a global activity of the clotting system.

In conclusion, in this study by the measuring of TG with the fully automated system ST Genesia we could confirm the role of ETP as a potential biomarker during chemotherapy supporting its inclusion in a model for the prediction of E-DR. To corroborate this data, the predictive score should be validated in an independent external cohort. However, the measurement of ETP through a standardized and automated system provides the basis for its use as a clinical test in the near future, supporting the clinicians in the selection of highest risk patients deserving targeted surveillance and specific treatment strategies.
