# Supplementary material for: Validation of the Role of Thrombin Generation Potential by a Fully Automated System in the Identification of Breast Cancer Patients at High Risk of Disease Recurrence
Source: TH Open. 2021 Feb 10;5(1):e56–65. doi: 10.1055/s-0040-1722609 (PMC7875677; doi:10.1055/s-0040-1722609)
Supplement: Supplementary file 1 — Supplementary Material [file 10-1055-s-0040-1722609-s200094.pdf]

Supplementary Material

HYPERCAN investigators:

- Immunohematology and Transfusion Medicine, ASST Papa Giovanni XXIII, Bergamo, Italy: Falanga Anna, Diani Erika, Gamba Sara, Giaccherini Cinzia, Gomez-Rosas Patricia, Marchetti Marina, Pesenti Marina, Russo Laura, Schieppati Francesca, Tartari J. Carmen, Verzeroli Cristina, Vignoli Alfonso
- Medical Oncology and Hematology, Humanitas Clinical and Research Hospital, Rozzano, Italy: Santoro Armando, Masci Giovanna
- Medical Oncology and Hematology, Fondazione IRCCS Istituto Nazionale dei Tumori, Milan, Italy: De Braud Filippo, Celio Luigi, Martinetti Antonia
- Medical Oncology, ASST Papa Giovanni XXIII, Bergamo, Italy: Tondini Carlo, Labianca Roberto
- Medical Oncology, Presidio Ospedaliero San Filippo Neri, Rome, Italy: Gasparini Giampietro, Sarmiento Roberta, Gennaro Elisabetta
- Medical Oncology, Azienda Ospedaliera San Giovanni-Addolorata, Rome, Italy: Minelli Mauro
- Medical Oncology, ASST Bergamo Ovest- Ospedale Treviglio Caravaggio, Treviglio, Italy: Barni Sandro, Petrelli Fausto, Ghilardi Mara
- Medical Oncology, IRCCS Istututo Tumori Giovanni Paolo II, Bari, Italy: Giuliani Francesco
- Internal Medicine and Medical Oncology, Policlinico San Marco, Gruppo San Donato, Zingonia-Bergamo, Italy: D'Alessio Andrea, Cecchini Sara

 © 2021. The Author(s).
